# Structure and dispersion of the conjugative mobilome in surface ocean bacterioplankton

**DOI:** 10.1093/ismeco/ycae059

**Published:** 2024-04-25

**Authors:** Javier Tamayo-Leiva, Jaime Alcorta, Felipe Sepúlveda, Sebastián Fuentes-Alburquenque, José Ignacio Arroyo, José Eduardo González-Pastor, Beatriz Díez

**Affiliations:** Biological Sciences Faculty, Pontificia Universidad Católica de Chile, Santiago 8331150, Chile; Center for Climate and Resilience Research (CR2), University of Chile, Santiago, Chile; Biological Sciences Faculty, Pontificia Universidad Católica de Chile, Santiago 8331150, Chile; Millennium Institute Center for Genome Regulation (CRG) , Santiago, Chile; Biological Sciences Faculty, Pontificia Universidad Católica de Chile, Santiago 8331150, Chile; Millennium Institute Center for Genome Regulation (CRG) , Santiago, Chile; Centro de Investigación en Recursos Naturales y Sustentabilidad, Universidad Bernardo O’Higgins, Santiago, Chile; Departamento de Matemáticas y Ciencias de la Ingeniería, Facultad de Ingeniería, Ciencia y Tecnología, Universidad Bernardo O’Higgins, Santiago, Chile; Biological Sciences Faculty, Pontificia Universidad Católica de Chile, Santiago 8331150, Chile; The Santa Fe Institute, Santa Fe, NM 87131, United States; Centro de Modelamiento Matemático, Universidad de Chile, IRL 2807 CNRS Beauchef 851, Santiago, Chile; Department of Molecular Evolution, Centro de Astrobiología (CAB), CSIC-INTA. Carretera de Ajalvir km 4, Torrejón de Ardoz 28850 Madrid, Spain; Biological Sciences Faculty, Pontificia Universidad Católica de Chile, Santiago 8331150, Chile; Center for Climate and Resilience Research (CR2), University of Chile, Santiago, Chile; Millennium Institute Center for Genome Regulation (CRG) , Santiago, Chile

**Keywords:** horizontal gene transfer, HGT, conjugation, mobile genetic elements, MGEs, mobilome, plasmids, relaxases, T4SS

## Abstract

Mobile genetic elements (MGEs), collectively referred to as the “mobilome”, can have a significant impact on the fitness of microbial communities and therefore on ecological processes. Marine MGEs have mainly been associated with wide geographical and phylogenetic dispersal of adaptative traits. However, whether the structure of this mobilome exhibits deterministic patterns in the natural community is still an open question. The aim of this study was to characterize the structure of the conjugative mobilome in the ocean surface bacterioplankton by searching the publicly available marine metagenomes from the TARA Oceans survey, together with molecular markers, such as relaxases and type IV coupling proteins of the type IV secretion system (T4SS). The T4SS machinery was retrieved in more abundance than relaxases in the surface marine bacterioplankton. Moreover, among the identified MGEs, mobilizable elements were the most abundant, outnumbering self-conjugative sequences. Detection of a high number of incomplete T4SSs provides insight into possible strategies related to trans-acting activity between MGEs, and accessory functions of the T4SS (e.g. protein secretion), allowing the host to maintain a lower metabolic burden in the highly dynamic marine system. Additionally, the results demonstrate a wide geographical dispersion of MGEs throughout oceanic regions, while the Southern Ocean appears segregated from other regions. The marine mobilome also showed a high similarity of functions present in known plasmid databases. Moreover, cargo genes were mostly related to DNA processing, but scarcely associated with antibiotic resistance. Finally, within the MGEs, integrative and conjugative elements showed wider marine geographic dispersion than plasmids.

## Introduction

Microorganisms can exchange DNA via horizontal gene transfer (HGT), thus quickly acquiring new phenotypes or traits [1]. HGT facilitates the dispersion of genes, such as antibiotic resistance genes (ARGs) [2, 3]. Marine systems have been associated with ARG dispersal due to effluent discharge into coastal areas [4, 5], as well as the dispersal of particles, such as plastics [6]. Furthermore, diverse metabolic functions [7], metal resistance [8], and several other gene functions [9, 10] are also HGT-borne.

In aquatic environments, models of gene flow dynamics have suggested that conjugation is the most active HGT process [11]. At least four different elements are associated with the conjugation process, enhancing its wide occurrence. (i) The nuclease relaxase MOB, which is responsible for specific cleavage of mobile DNA, covalent bonding and initiating rolling-circle replication to produce ssDNA for conjugative transfer [12]. (ii) Cell-to-cell contact mediated by the type IV secretion system (T4SS) that facilitates DNA delivery and ensures its integrity [13]. (iii) The possibility that phylogenetically distant bacterial lineages, even across domains, exchange DNA [14]. And finally, (iv) the arrangement of these elements in mobile genetic elements (MGEs), which can facilitate gene dispersal across bacterial communities providing a favorable genetic context (i.e. operons, regulators, etc.) [15].

The best-studied conjugative MGEs, which are transferable MGEs via the host’s conjugation machinery, include conjugative plasmids, transposons, and integrative and conjugative elements (ICEs). Self-conjugative MGEs encode all the necessary conjugative machinery [16, 17]. Interestingly, MGEs can also act as helpers and supply *in trans* all the necessary machinery for non-self-conjugative element transfer [18]. Thus, trans-acting activity between MGEs can enhance element conjugation and persistence [19].

Since the late 2000s, studies have referred to the entire collection of MGEs within a population, community, or environment as the mobilome [14]. In the marine system, mobilome studies are important, given bacterial dispersal over large geographic regions [20–22]. Examples of highly dispersive MGEs, such as class I integrons [23] or the marine plasmid pLA6 [24], have been reported in this system. However, whether the mobilome exhibits the same macroecological pattern as microbial taxa remains unresolved.

The present work represents an in-depth study of the conjugative marine mobilome that aims to (i) identify and expand our knowledge of molecular signatures, dispersion of cargo genes and MGEs and (ii) better understand mobilome similarity across marine bacterial communities over large marine areas. For this purpose, we selected 45 metagenomes from the TARA Oceans expedition [25, 26], representing the bacterial free-living size fraction (0.2 – 3 μm) of marine surface waters, which have been described as the most dynamic communities [27]. We used an *in silico* approach to identify the most abundant conjugative elements in the marine mobilome through marker genes, such as relaxases and the type 4 coupling protein ATPase (T4CP). The results demonstrate a wide geographical dispersion of MGEs in marine bacterioplankton communities from most oceanic regions, except for the Southern Ocean, which shows a less connected community and mobilome composition.

## Materials and methods

### TARA oceans sample data selection and 16S rRNA gene amplicon processing

**Figure 1:**
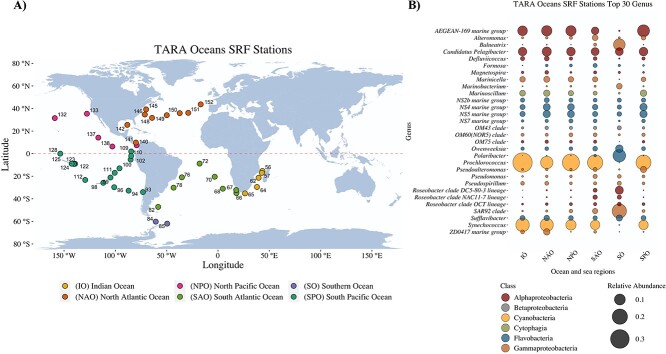
Stations selected for this study from the TARA oceans dataset. Size-fraction 0.22-3.0 μm. A) Samples selected and enumerated by station ID. Stations are colored by ocean regions as follows: South Pacific Ocean (SPO), North Atlantic Ocean (NAO), Southern Ocean (SO), North Pacific Ocean (NPO), South Atlantic Ocean (SAO), Indian Ocean. B) Top 30 genera in marine surface water. Top 30 genera selected as the total counts for each genus. The color represents the taxonomic class; bubble sizes represent the average relative abundance of each genus in each ocean and sea region.

Assembled metagenomes from 45 stations targeting the free-living bacteria size fraction (0.2 – 3 μm) of the surface layer (5 to 12 m depth) collected during the TARA Oceans expedition were downloaded from the European Nucleotide Archive (ENA Project accession PRJEB1787). These stations included the Indian Ocean (n = 5), South Atlantic Ocean (n = 8), Southern Ocean (n = 2), South Pacific Ocean (n = 15), North Pacific Ocean (n = 6), and North Atlantic Ocean (n = 9) (Fig. 1A). Supporting information was compiled for the environmental data and associated biological and oceanographic conditions at these 45 stations from the official dataset that is freely available on the TARA Oceans companion website, Tables W1–W8 (http://ocean-microbiome.embl.de/companion.html).

Raw sequences of the 16S rRNA gene were also downloaded from the official companion website and the 16S rRNA miTAG dataset (http://ocean-microbiome.embl.de/data/16SrRNA.miTAGs.tgz). The selected 45 samples from surface waters were quality filtered using the q2-demux plugin implemented in the Qiime2 (qiime2-2019.10) pipeline [28, 29]. The paired-end sequences were trimmed and joined using DADA2 [30] to obtain amplicon sequencing variants (ASVs). Taxonomy of 16S rRNA ASVs was assigned using the q2-feature-classifier with the SILVA132 database [31] via the “classify consensus vsearch” method [32]. ASV analyses were conducted with phyloseq [33] and ampvis2 [34] R packages. Visual support was employed with ggplot2 [35] and ggmap [36] R packages. Bacterioplankton diversity analysis of the 16S rRNA gene sequences ASVs was performed with the stats [37] and vegan [38] packages in R.

### Mobile genetic element read recruitment, normalization, and coverage estimation

Generation of non-redundant (NR) contigs was based on available assemblies from the TARA Oceans dataset, which were considered to be optimal assemblies (ENA Project accession PRJEB1787) [25], and on the TARA metagenome-assembled genome (MAG) collection (https://doi.org/10.6084/m9.figshare.4902923.v1) [39]. Contigs detected here as potential MGEs, retrieved from the 45 surface samples (Fig. 1A), were clustered with cd-hit version 4.7 [40] at 95% average nucleotide identity across 90% of each contig length. The non-redundant MGE contigs were then mapped with Bowtie2 version 2.3.4.1 [41] against the previously compiled collection of 957 NR MAGs [39] with the —al-conc parameter. To estimate contig sequence read recruitment and coverage, clean sample reads were then downloaded from the EBI TARA Oceans repository (ENA Project accession PRJEB1787) and mapped to the NR contigs with Bowtie2 as above. Read counts were normalized using the library size scaling factor trimmed mean of M-values “TMM” function of edgeR [42]. Subsequently, scaled samples were normalized with the transcript per million method. MGEs with at least 80% of the contig length and ≥ 1× coverage were classified as “detected” in a metagenome, and only sequences detected with a threshold of at least six metagenomes were considered as “highly dispersed” MGEs.

### Mobile genetic element identification and plasmid references

Conjugative genes were searched using hidden Markov models (HMM) [43]. For putative MGE identification, a collection of 16 HMMs with 12 relaxases (i.e. PF01076, PF03389, PF03432, PF04899, PF05713, PF07057, PF07514, PF07515, PF08751, PF13814, PF16932, and PF17511) and four T4CP models (i.e. PF02534, PF07916, PF10412, and PF12696) were selected from the Pfam database (Pfam 32.0) (Table S1). The search was performed using the hmmsearch (HMMER3) function with the “trusted cutoff” setting for each HMM model [44] against the total collection of predicted coding sequences (CDSs), which was generated using Prodigal version 2.6.3 with the metagenome option (—meta) [45]. After contig identification by a positive match against one of the 16 models, a second filter was applied to enhance the probability of the match originally being an MGE. Contigs with at least one predicted relaxase or a T4CP, plus other putative T4SS proteins were retained for further analysis.

The taxonomic assignation of the MGEs was accomplished through taxonomic annotation with the co-assembled MAGs collected by Delmont *et al.* [39] and the genetic distance (i.e. kmers) obtained from the webserver of the Plasmid Sequences database (PLSDB version 2020-06-29; https://ccb-microbe.cs.uni-saarland.de/plsdb/) using the integrated mash sketch software version 2.1.1 with the max_dist of 0.4 [46]. Plasmid sequences from the PLSDB were filtered by completeness, and those annotated as “complete” (9693 total) were downloaded from RefSeq by their accession number. The PLSDB sequence features (i.e. taxonomy, size, Inc and Rep type, and topology) were also used to compare with the MGE sequences detected in this study (Fig. S1).

For the classification of conjugative, mobilizable, and non-conjugative elements, the following criteria was applied. (i) The sequence was classified as a “putative conjugative system” or “conjugative” [47] if it carried a relaxase, a T4CP and a T4SS ATPase (e.g. VirB11, VirB4, TraC, and TrwD ATPase) [48]. T4SS ATPase sequences were identified by the Pfam Model T2SSE (i.e. PF00437). (ii) The sequence was classified as “mobilizable” if it carried a detectable relaxase, but an incomplete conjugative system (i.e. relaxase, T4CP, and T4SS ATPase). Finally, (iii) the sequence was classified as “non-conjugative” if it carried an incomplete conjugative system without a detectable relaxase. It is important to note that the sequences classified here as non-conjugative, in a cellular context, could be mobilized by interaction with other T4SSs [16, 49]. Therefore, this is an operational definition for the purpose of this analysis. An additional criterion was then applied to classify the putative MGEs by sequence type, namely as an integrative and conjugative element (ICE), plasmid or secretion system. For identifying integrases in the MGE sequences, specific models for tyrosine and serine recombinases were used (serine: PF00239, PF07508; tyrosine: PF00589). If the element reported a match with any of these models, the sequence was classified as an “ICE.” If the sequence did not match any tyrosine or serine recombinase but carried a relaxase, the sequence was classified as a “plasmid”. Finally, if the sequence did not have a relaxase, nor a tyrosine or serine recombinase, the sequence was classified as a “secretion system”.

### Functional annotation of mobile genetic elements

Annotation of relaxase MOB families was performed with the HMM curated models provided by MOBscan, (i.e. MOBfamDB; https://castillo.dicom.unican.es/mobscan_about/MOBfamDB.gz). The database includes 11 curated HMMs to annotate nine relaxase MOB families [50]. The search of MOB families was performed with hmmscan and matches with ﻿*P*-value <.001 ﻿were considered as positive.

Functional annotation of CDSs from MGE contigs was performed using two different databases. First, the Pfam 32.0 database [51] and the hmmscan function with the “trusted cutoff” setting for each HMM model was used. Subsequently, the results were complemented with the COG classification generated by the eggnog-mapper web server (http://eggnog-mapper.embl.de; Set-up: e-value <0.001, min. Coverage of 50%) with a stricter minimum coverage than the default option (20 %) [52]. Clustering of Pfams model distribution across MGE size subgroups was performed with the hclust function of the R stats package version 4.2.2 [37] using the Canberra distance.

### Alpha and beta diversity analyses

Alpha diversity was measured as the number of distinct plasmids in each sample (MGE richness) or the number of distinct 16S rRNA ASVs in each sample using the Shannon’s diversity index. Beta diversity using the Jaccard index [53] was employed to measure the compositional similarity between samples (MGEs or ASVs). MGE richness was correlated with the environmental variables and the observed richness of functions (OG Richness) (Tables W1-W8) using simple linear regression. The relationship between community similarity and geographic distances or “distance decay” was investigated via simple linear regression analysis between samples log Jaccard similarity index and the geographic distance (i.e. Haversine distance) among sites. A permutational multivariate analysis of variance (PERMANOVA), using the adonis2 function of the vegan [38] package with 9999 permutations, was used to investigate the difference among the 45 samples based on alpha (Shannon index) or beta (Bray–Curtis distance) diversity, and its association with oceanographic or environmental factors.

## Results

### Oceanic regions drive prokaryotic community structures

Free-living (0.2–3 μm) microbial communities in surface metagenomes from 45 stations of the oceanic regions of the TARA Ocean database were studied (Fig. 1A). In these communities, the top 30 genera (1.39% mean cumulative percent abundance, and 27.1% of the maximum cumulative percent abundance observed) were dominated by Alphaproteobacteria of the SAR11 clade (*Candidatus* Pelagibacter), AEGEAN-169 marine group, Cyanobacteria (*Prochlorococcus* and *Synechococcus*), and several genera of the class Flavobacteria (Table S2). Although the presence of these taxa was homogenous across all oceanic regions, an exception to this general pattern was observed in the Southern Ocean, where Cyanobacteria were absent. In contrast, *Polaribacter*, *Roseobacter*, *Balneatrix*, and Alteromonadales of the SAR92 clade were present there in higher relative abundance (Fig. 1B). PERMANOVA results (Table S3) show that the oceanographic region is the most significant factor affecting species richness and community structure (R2 = 0.537 (﻿*P*-value .0003), and R2 = 0.376 (﻿*P*-value .001), respectively).

### Mobilizable elements dominate the marine MGEs communities

After analysis of the bacterioplankton composition, we further identified putative conjugative MGEs in the 45 metagenomes. From a total collection of 13 911 642 contigs, 1788 (0.012%) reported the presence of at least one match against the HMM models for MGEs (Table S1). However, after applying a complementary selection filter based on the presence of more than one genetic marker, 313 non-redundant (NR) contigs remained for further analyses (see Materials and methods). The sequences were then mapped against the co-assembled MAGs collected by Delmont *et al.* [39]. The result shows that 94 of the 313 NR sequences were part of 66 longer contigs in the MAG collection, reducing the number to 285 NR sequences. These remaining NR sequences were grouped into seven sequence size groups ≤1 kb (n = 76), 1–5 kb (n = 75), 5–10 kb (n = 33), 10–15 kb (n = 20), 15–50 kb (n = 44), 50–200 kb (n = 29), and > 200 kb (n = 8). In total 102 sequences were longer than 10 kb (Table S4). Of the 76 sequences < 1 kb, only one relaxase gene was detected, therefore only the 209 NR sequences >1 kb were further analyzed. Taxonomic analysis of the sequences showed that 124 of the 209 MGE NR sequences were assigned to a defined taxonomic group. The dominant phylum for all sequence size groups was Proteobacteria (44% in sequences >1 kb), followed by Actinobacteria (6% in sequences >1 kb), whereas several unassigned sequences were present at sizes between 1 and 5 kb (Fig. S2).

**Figure 2:**
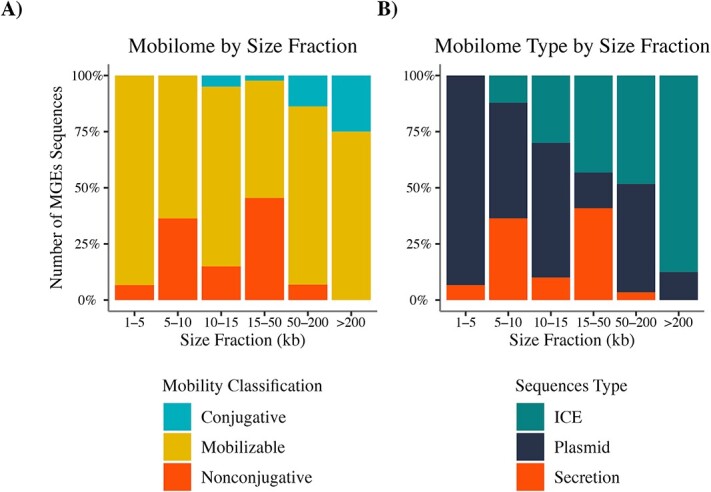
Mobile genetic element (MGE) size distribution by mobilization and MGE type. MGE sequence size distribution by a) mobilization type or B) MGE type.

After identifying the taxonomy of putative conjugative MGEs, a classification based primarily on their mobility (i.e. conjugative, mobilizable, and non-conjugative) was performed, and then complemented by a classification based on the MGE type (i.e. plasmid, ICE, secretion system; see Materials and Methods). Mobilizable elements were dominant for all contig sizes, while conjugative sequences were identified in only those >10 kb (Fig. 2). Of the total 209 sequences reported, 8 were classified as conjugative (3.8%), 159 as mobilizable (76.1%), and 42 as non-conjugative (20.0%) (Fig. 2A, Table S5). The MGE classification scheme indicates that plasmid sequences dominated the sequence size fraction between 1 and 15 kb. In contrast, the ICE type dominated in the largest contigs (Fig. 2B, Table S5). Statistical analysis of variance between groups corroborates that ICE sequences were significantly more represented than other element types in the larger contigs (Kruskal–Wallis test *P* = 3.1e-15) (Fig. S3).

**Figure 3:**
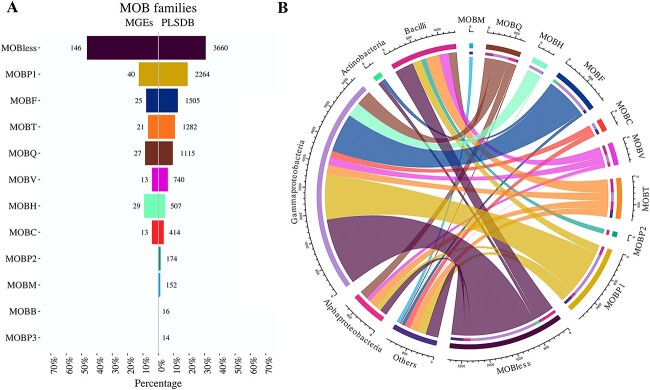
MOB classification. A) MOBscan classification of relaxase sequences from marine bacterioplankton MGEs (this study) and reference PLSDB sequences. B) MOBscan taxonomic classification. MOB type classification of PLSDB sequences by taxonomic level.

Lastly, due to the broad-host range of some MGEs, an approach was also applied to identify possible taxonomic boundaries of the MOB families. Both, the marine MGEs and the PLSDB relaxases were classified using the MOBscan collection [50], comprising curated HMM models of MOB families. The results show a higher proportion of sequences in the marine mobilome with unclassified relaxases (i.e. MOBless) and MOBH class relaxases relative to that observed in the PLSDB collection (Fig. 3A). Conversely, both datasets showed MOBP1 and MOBF as the most abundant models. The marine MGEs relaxases also showed that the most represented MOB types (MOBless and MOBP1) are taxonomically assigned to Gammaproteobacteria, while this class also shows most of the representation of the MOBH and MOBF families (Fig. 3B).

### Nucleases complementarity and redundancy in marine MGEs

Within the mobilizable sequences, 28 elements carried more than one relaxase gene, and 17 had the relaxase pair comprising Pfam model MobC (PF05713) and Relaxase (PF03432) (Table 1). These relaxase pairs were contiguous genes in all detected contigs. These sequences were predominantly of the phylum Proteobacteria (10 sequences). Among them, three were affiliated (genetic distance ≤20%) with PLSDB plasmids (pP43BP1 (4,90 bp), MGE 049; pA3H7 (6788 bp), MGE 053; and pPAMC27889 (16 905 bp), MGE 093) from genomes of the family Moraxellaceae (genus *Psychrobacter*). Another alignment with lower genetic distance (≤ 5%) was affiliated with a plasmid (pR997 (85.368 bp), MGE MAG 057) of the genus *Proteus* (Gammaproteobacteria), whereas the holder MAG was affiliated with the class Alphaproteobacteria.

**Table 1:** Mobile genetic elements (MGEs) in the ocean microbiome, with more than one relaxase/MOB gene.

Sequence ID	Classification	Type	Lenght (kb)	MOBscan	PFAM Models Name	CDS	Phylum	Class	Order	Family	PLSDB Reference	Accession ID	Distance^*^
MGE_031	Mobilizable	ICE	22.592	MOBP1	MobC, Relaxase	12, 11	Actinobacteria	Actinobacteria	Micrococcales	Microbacteriaceae	pA10BH1	MN657133.1	24%
MGE_049	Mobilizable	Plasmid	1.994	MOBP1	Relaxase, MobC	1, 2	Proteobacteria	Gammaproteobacteria	Pseudomonadales	Moraxellaceae	pP43BP1	NC_019276.1	20%
MGE_053	Mobilizable	Plasmid	6.73	MOBP1	Relaxase, MobC	3, 4	Proteobacteria	Gammaproteobacteria	Pseudomonadales	Moraxellaceae	pA3H7	MN657087.1	13%
MGE_070	Mobilizable	Plasmid	1.19	MOBP1	MobC, Relaxase	2, 1							
MGE_086	Mobilizable	Plasmid	3.771	MOBP1	MobC, Relaxase	5, 6							
MGE_093	Mobilizable	Plasmid	4.161	MOBP1	MobC, Relaxase	1, 2	Proteobacteria	Gammaproteobacteria	Pseudomonadales	Moraxellaceae	pPAMC27889	NZ_CP014946.1	20%
MGE_163	Mobilizable	ICE	231.544	MOBP1, MOBF, MOBF, MOBT	TrwC, TrwC, Relaxase	183, 119, 114, 108							
MGE_164	Mobilizable	ICE	219.912	MOBP1	MobC, Relaxase	153, 154							
MGE_169	Mobilizable	ICE	423.718	MOBT, MOBP1	Relaxase, MobC	397, 408, 409							
MGE_170	Mobilizable	ICE	55.946	MOBP1	MobC, Relaxase	45, 44	Cyanobacteria		Synechococcales	Synechococcaceae	Unnamed	NZ_CP013999.1	30%
MGE_175	Mobilizable	ICE	11.696	MOBF, MOBT	TrwC	9, 6	Proteobacteria	Alphaproteobacteria	Rhodobacterales	Rhodobacteraceae	pTHAF9_c	NZ_CP045407.1	30%
MGE_187	Mobilizable	Plasmid	1.423	MOBP1	Relaxase, MobC	2, 1							
MGE_210	Conjugative	ICE	47.361	MOBP1	Relaxase, MobC	17, 18	Cyanobacteria		Nostocales	Nostocaceae	pNPUN03	NC_010630.1	30%
MGE_220	Mobilizable	Plasmid	4.027	MOBP1, MOBQ	MobA_MobL	3, 2							
MGE_238	Mobilizable	Plasmid	2.938	MOBP1	Relaxase, MobC	2, 3							
MGE_276	Mobilizable	Plasmid	2.62	MOBP1, MOBQ	MobC, Relaxase	1, 2	Proteobacteria	Alphaproteobacteria	Rhodobacterales	Rhodobacteraceae	pCAI42A	NZ_CP012662.1	16%
MGE_MAG_001	Mobilizable	ICE	124.175	MOBQ	MbeD_MobD, MobA_MobL	104, 124	Proteobacteria	Alphaproteobacteria	Rhizobiales	Rhizobiaceae	pAt	NC_003064.2	30%
MGE_MAG_014	Conjugative	ICE	112.332	MOBP1	Relaxase, MobC	74, 75	Proteobacteria						^*^^*^
MGE_MAG_015	Mobilizable	ICE	26.136	MOBP1	MobC, Relaxase	18, 17	Proteobacteria						^*^^*^
MGE_MAG_023	Mobilizable	ICE	56.146	MOBP1	Relaxase, MobC	2, 1	Bacteroidetes	Cytophagia	Cytophagales		Unnamed	NZ_CP013999.1	30%
MGE_MAG_024	Mobilizable	Plasmid	206.968	MOBT, MOBH	TraI_2, TraI_2_C	5, 134	Proteobacteria	Gammaproteobacteria	Legionellales		pPSEM90-4	NZ_CP033941.1	30%
MGE_MAG_027	Mobilizable	ICE	208.141	MOBP1, MOBT	MobC	151, 152, 137	Proteobacteria	Gammaproteobacteria	Alteromonadales	Alteromonas			^*^^*^
MGE_MAG_038	Mobilizable	ICE	309.926	MOBP1, MOBT, MOBT, MOBP1	Relaxase	222, 268, 52, 1	Proteobacteria	Alphaproteobacteria					^*^^*^
MGE_MAG_039	Mobilizable	Plasmid	148.43	MOBV, MOBQ, MOBC	MobA_MobL	137, 133	Proteobacteria	Alphaproteobacteria					^*^^*^
MGE_MAG_046	Mobilizable	Plasmid	65.975	MOBF, MOBP1	Relaxase, MobC, TrwC	19, 20, 36	Proteobacteria	Alphaproteobacteria	Rhodobacterales	Oceanicaulis	pMOL30	NC_007971.2	30%
MGE_MAG_057	Mobilizable	ICE	85.238	MOBT, MOBH	TraI_2	60, 65	Proteobacteria	Alphaproteobacteria			pR997	NZ_KY433363.1	5%
MGE_MAG_060	Conjugative	ICE	1019.813	MOBT, MOBT, MOBT, MOBT, MOBT	Replic_Relax	551, 832, 247, 320, 327, 548	Proteobacteria	Gammaproteobacteria	Alteromonadales		pDJL3	NZ_CP025962.1	30%
MGE_MAG_063	Mobilizable	ICE	60.15	MOBF, MOBT	TrwC	28, 12	Actinobacteria	Actinobacteria	Actinomycetales	Microbacterium			^*^^*^

Taxonomy: ^*^Genetic distances calculated as mash distances with PLSDB sequences in the PLSDB web server [46]; ^*^^*^Taxonomy assigned with TARA MAGs collected by Delmont et al., [39].

Most of the MGEs (27 sequences) in the non-conjugative category (41 sequences) were not taxonomically identified. However, among those identified, Proteobacteria dominated (10 sequences). A search for other putative nucleases in this group was performed to check whether these elements carried nucleases that could act as a substitute for the relaxase function. In 23 of the 41 MGEs classified as non-conjugative, several nucleases were identified, and were dominated by helicases (Viral - Superfamily 1 - RNA helicase, PF01443; UvrD/REP helicase, PF00580; Helicase_C, PF00271; helicase HerA (DUF87), PF01935). The archaeal helicase HerA (DUF87) was the dominant model (detected in 16 of 41 sequences). Integrases and transposases (Integrase 1, PF12835; Transposase IS116/IS110/IS902 family, PF02371) were also frequently found. In addition, other enzymes found within the MGEs were topoisomerases (DNA topoisomerase, PF01131) and endonucleases (Res subunit of type III endonucleases, PF04851). Even though these sequences were assigned to non-conjugative elements, nine shared identities with plasmid references (Table S6). An interesting case among the non-conjugative MGEs was MGE 128, encoding 10 T4SS genes and a viral RNA helicase (PF01443), but no canonical relaxase. Finally, four MGE sequences classified as mobile belonged to ICEs and four others belonged to plasmids. These MGE sequences carry the model relaxase (PF03432), T4SS_TraI (PF16932) and MobC (PF05713). At the same time, the two largest sequences (more than 200 kb) belonged to ICEs and showed several integrases and relaxases gene copies (i.e. MGE MAG 041; 060) (Table S7). From eight mobile sequences, five shared a genetic distance of <30% with PLSDB reference sequences of the Proteobacteria, Verrucomicrobia, and Cyanobacteria phyla.

### Functional diversity of mobile genetic element genes

**Figure 4:**
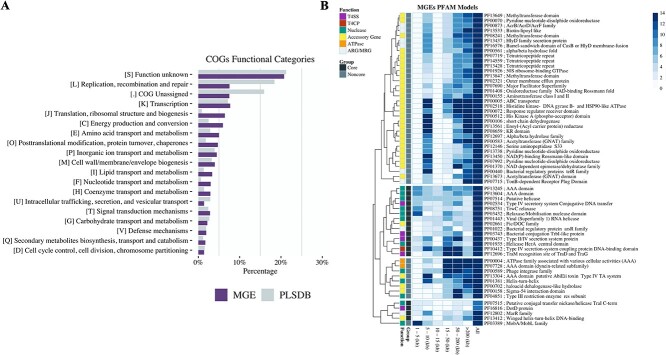
Functional annotation of detected marine bacterioplankton mobile genetic elements (MGEs). A) COG functions grouped by MGE sequences (this study) and reference PLSDB sequences. B) Heatmap of the Pfam models within the top 2% richness and core Pfam models. Groups are represented if the Pfam model belongs to the top 2% or to the core models. The black squares represent sequences detected in all MGE fraction sizes. Grey squares represent Pfam models within the top 2% richness. The functions are group by color accordingly to the molecular function of the model. The heatmap rows are ordered according to the hierarchical clustering in the left calculated by the Canberra distances between the pfam profiles.

To complement the annotation and characterization of all detected MGEs, we also analyzed their functional diversity based on two strategies. First, we compared the COG functional assignments of the curated plasmids from the PLSDB versus those observed in our collection of MGEs. The results show a similar distribution of COG groups between the two datasets, where S (unknown function), L (replication, recombination, and repair), and K (transcription) groups were the predominant. In contrast, V (defense mechanism), which mainly includes ARGs, had little representation in both datasets (Fig. 4A).

As a second strategy, we analyzed the most frequent Pfam domain patterns for the predicted CDSs in the 209 NR sequences. Annotations of the 7539 CDSs with the Pfam database identified 5136 CDSs, reporting 2682 unique accessions. Next, Pfam models with the most detected hits (abundance value in the top 2%) were targeted for functional analysis. The results identified 18 core Pfam models (i.e. detected in all size divisions >1 kb) and 43 non-core models (Fig. 4B; Fig. S4). Among the core Pfam models retrieved, most were related to conjugative functions, such as relaxases, helicases, T4CP, T4SS proteins, and a chimeric secretion system of the DotD Type IVB protein. Other core models not directly related to conjugation were the multiple antibiotic resistance repressor MarR and the arsenic-responsive repressor ArsR. The abundance of these core functions was heterogenous across size groups. Other models previously related to MGEs were also abundant within the non-core models, such as the phage integrase model and a type IV toxin–antitoxin resistance system. A higher abundance of non-T4SS-related accessory functions was observed with increasing sequence size. A higher frequency of detection was also observed for functions such as oxidoreductases, methyltransferases, membrane efflux system, metal resistance, TetR family and other ARGs-coupled models.

### Drivers of mobile genetic element community structure

**Figure 5:**
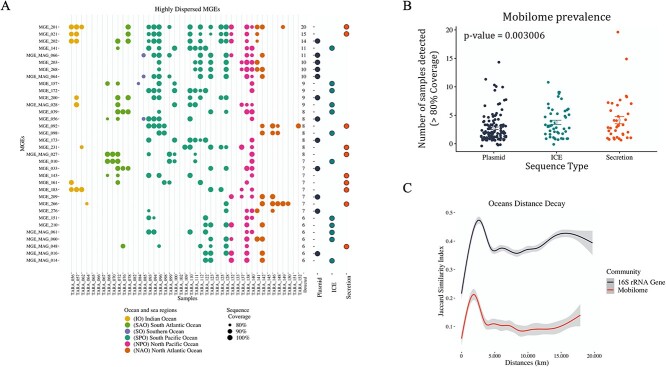
High dispersion and coverage of mobile genetic elements (MGEs) throughout marine surface bacterioplankton. Stations selected for this study are from the TARA oceans dataset, size-fraction 0.22 - 3.0 μm. A) MGE sequences with high dispersion (80% sequence length with ≥1 coverage in ≥6 metagenomes). Bubble sizes represent the percentage of coverage for each MGE sequence, only values ≥80% coverage were included in the graphic representation. Stations are colored by ocean regions. B) Mobilome prevalence by MGE type. Color represents the classification of each MGE. C) Distance decay. Distance decay was computed as the linear regression of the Jaccard similarity matrix (mobilome and 16S rRNA gene) with the linear geographic distance matrix (m).

To study the diversity and geographical dispersion patterns of MGEs across bacterioplankton communities, the richness and prevalence of the 209 MGE NR sequences (> 1 kb) were analyzed in the 45 metagenomes. Richness analysis of the observed MGEs showed no specific association with any oceanographic location (Fig. S5A). However, a significant but weak association (R^2^: 0.260; *P* < 10^-3^) was detected between the observed functional richness of orthologous genes (OG Richness) and the richness of MGE sequences (Fig. S5B). Conversely, prevalence analysis showed a wide geographical dispersion of MGEs with high identity across the surface bacterioplankton communities (Fig. 5). Among the 34 sequences showing the highest dispersion, 22 shared a genetic distance (≤ 30%) with the reference sequences and were dominated by the phylum Proteobacteria (56%) (Table S8). Among the most dispersed sequences were MGE 201 (43 kb) and MGE 021 (6 kb), both of which were identified as non-conjugative elements with no detected relaxase, followed by the mobilizable plasmid MGE 202 (11 kb) and the ICE sequence MGE 141 (107 kb) (Fig. 5A). In addition, some reference sequences were mapped and found with wide oceanic prevalence. This was the case for plasmid pR997 (85 kb) from *Proteus mirabilis*, plasmid p123 (122 kb) from *Vibrio tubiashii*, and plasmid pMSPYR102 (24 kb) from *Mycobacterium gilvum*.

Of all the marine ocean regions included in the present study, the Southern Ocean represents the region with the most differences in terms of MGEs. Although most sequences were detected in more than one region, the majority went undetected in the Southern Ocean. These surface waters shared only four widely dispersed MGEs (three belonging to Proteobacteria and one to Actinobacteria) with other oceanic regions. Subsequently, a sample prevalence analysis grouped by MGE type or size was performed (Fig. 5B). The results showed no correlation between the sequence size or the number of metagenomes in which were detected (Kruskal–Wallis test *P* > .05). However, a significant difference in dispersion (number of samples presence) was observed between ICEs and plasmids (Kruskal–Wallis test *P* = .0088), revealing that chromosome-integrated elements, such as ICEs and secretion systems, undergo greater geographical dispersion than plasmids (Fig. 5B). In the case of the more geographically dispersed MGEs, COG functions, such as I (lipid metabolism), P (inorganic transport), T (signal transduction), and Q (secondary metabolism), were more abundant than in the rest of the oceanic mobilome, while V (defense mechanism) remained in the same proportion (Fig. S6). To identify whether the marine mobilome undergoes greater geographical dispersal than the bacterial taxa, we further performed a distance decay analysis that showed a lower similarity between the mobilome than the marine microbiome (Fig. 5C). Moreover, this analysis showed a weak correlation between ecological distance (i.e. Jaccard’s similarity index) and geographical distance (i.e., Haversine distances) (microbiome R^2^ = 0.003, *P* = .039; mobilome R^2^ = 0.04, *P* ≤ .001). These results confirm that less geographical dispersion occurs for MGE sequences than for the microbiome (i.e. 16S rRNA ASVs) (Fig. 5C).

## Discussion

In the present study, we must first address the limitations of the current methodology for analyzing MGEs, such as the difficulties in recovering complete high molecular weight sequences, due to the use of short read sequencing [54]. This is particularly relevant for detecting conjugative sequences that tend to be larger than mobilizable sequences [55, 56]. Likewise, there are difficulties in detecting sequences associated with undescribed relaxases, or conjugative sequences that do not share the classical structure of a self-conjugative or mobilizable MGEs [49, 55, 57]. Moreover, to understand the contribution to HGT and the dispersion of MGEs in the marine environment, it is crucial to consider their high mosaicism and to avoid overrepresentation of sequences due to fragmentation or detection of bacterial chromosomes [14, 47, 58, 59]. Consequently, a filter based on conjugative capacity (more than one marker gene), MGE type and coverage threshold was applied for more reliable detection of sequences in the metagenomes [10, 14, 47, 58].

### Characteristic marker gene combinations for MGEs in the marine mobilome

In this work, a high number of recovered sequences lacked a complete conjugative system, which could be explained by technical limitations (e.g. incomplete sequences or low coverage) due to short-read sequencing [60], together with high sequence variation among related MGEs [14, 61]. Another explanation could be a high dependence on trans-acting conjugation, which has been observed in other studies [47, 55, 59]. However, functional, and minimally mobilizable elements exist and have been characterized only by the presence of a relaxase gene and the origin of transfer (*oriT*) [55]. Despite these classifications, there are reports of non-conjugative elements capable of recruiting relaxase genes from other sequences in a process called *oriT*-mimicry [16, 57].

Typically, T4SS proteins are more abundant than relaxases within bacterial genomes and MGEs [47], which is consistent with a higher abundance of contigs with a single T4SS protein compared to those with T4SS in addition to relaxases. Furthermore, the high mosaicism and chimeric features of T4SS is supported by the fact that the system reportedly tends to localize to multiple and distant loci, and that it interacts with other secretion systems [62, 63]. The above is consistent with the higher abundance of incomplete T4SS found in our study. In contrast, the identification of conjugative MGEs could also have been hampered by the presence of unclassified relaxase sequences or those belonging to new relaxase-like families [55, 61, 64].

To date, nine MOB families have been described, six of which belong to the HUH endonuclease family (MOBV, MOBP, MOBB, MOBQ, MOBF, and MOBH). The other three are non-HUH families, namely MOBT (Rep_trans, RCR initiation proteins), MOBC (restriction endonuclease), and MOBM (Tyr recombinase) [12]. Of all the MOB families, MOBP and MOBF are prevalent in plasmid databases [61] as also in this study. The “Relaxase” (PF03432) and “TrwC” (PF08751) models were assigned to MOBP1 (MOBP family) and MOBF, respectively, in agreement with both our results and the MOBscan classification [50]. Meanwhile, the Pfam model “MobC” (i.e. PF05713) of MobC-like family sequences was not assigned to any MOBscan reference model. MOBC and MOBP are important relaxase protein families commonly found in elements with a broad host spectrum [47].

Our reults frequently found the pair configuration between MOBC and MOBP relaxases. This could suggest that these elements incorporate different types of relaxases to enhance their potential as substrates for conjugative systems provided *in trans*. MOBC relaxase has been described to assist in the mobilization of MGEs in the presence of a second relaxase *in cis* [65]. Furthermore, sequences with multiple relaxase genes are rare and, while the MOBP family is frequently detected among plasmids, detection of MOBC is unusual [61, 66]. Therefore, configuration of the MOBC–MOBP relaxase pair as a stochastic process is expected to be rare among marine MGE sequences, raising the possibility that this configuration has undergone evolutionary selection. This assumption is also supported by the presence and distribution of the geographically widespread marine plasmid pLA6 from the *Roseobacter* group, where the MOBC–MOBP pair was also observed [24].

Non-conjugative sequences in the marine mobilome (i.e. MOBless or undetected relaxases) frequently carry the T4CP–ATPase protein pair. Non-conjugative organization can be recognized by the requirement for the presence of a trans-acting conjugative relaxase [49], or an unannotated relaxase-type nuclease [16, 57]. According to the Pfam database, the HerA model of the archaeal helicase identified in this study can also be detected in the TraC protein (i.e. ﻿VirB4 ATPase), which is conserved among T4SS and required for pilus biogenesis [55]. Also detected in this study, the UvrD-helicase has been described as one of the genes involved in chromosomal compensatory adaptation (i.e. mutations that reduce the MGE metabolic burden) for the host-plasmid in *Pseudomonas aeruginosa* [67]. Therefore, the detection of UvrD in marine MGE sequences could be related to the adaptation of these molecules. However, a more detailed study in cellular models is needed to assess their function, which could depend not only on the host but also on the environment [7, 67].

The presence of sequences with T4SS and undetected relaxases could also be associated with the recruitment of T4SS components for new biological functions, as is frequently observed in bacterial secretion systems [63]. Therefore, the abundance of T4SS proteins should not be associated exclusively with a conjugative purpose in marine bacterioplankton [63].

### Functional contribution and geographic dispersion of the marine mobilome

Functional diversity analysis of the marine mobilome showed that the COG category V - Defense mechanisms, in which ARGs are included, was almost undetected at all investigated geographic locations (Fig. 4), even among the most highly distributed sequences (Fig. S7). Even further, the MarR family pfam model (PF12802) was found in the core mobilome functions, which is characterized as a repressor of the multiple antibiotic resistance Mar systems, possibly having regulation or interference roles with the antibiotic resistance of the host as seen with other traits [68]. In contrast, functions associated with molecular DNA processing were dominant, similar to that found in a previous analysis of the NCBI plasmid database [69]. The function and distribution of cargo genes found in different marine regions, including the Southern Ocean, suggest an evolutionary selection of sequences with replicative and DNA-integrative functions, but less association with ARG geographical dispersal. This is consistent with the observations of Cuadrat *et al*. [70], who also analyzed the TARA Oceans dataset, in that most ARGs were found primarily in chromosomal sequences rather than plasmids.

The surface marine environment is a highly dynamic system in which bacterial adaptation and community mixing are common processes [5, 22, 71]. Hence, it is possible to observe marine taxa with known global distribution, such as SAR11, Flavobacteriales and Rhodobacterales [21, 25]. Consistent with the literature, the Southern Ocean is an isolated region where the conditions shape bacterioplankton communities differently from other oceanic regions (including the Arctic Ocean), as well as driven by seasonal eukaryotic phytoplankton blooms, which boost primary production in this oceanic region [72]. All of the above give rise to unique cellular OTUs [21], as also observed in the mobilome revealed by this study.

The marine conjugative mobilome described here was mainly associated with Proteobacteria, which agrees with the dominance and wide dispersion of this phylum in the marine system [21, 22, 25, 73]. However, there are also other previous oceanic examples of MGEs with highly conserved identity and dispersal across large geographic areas, such as the ICE class I integron, the ﻿*Vibrio cholerae*-associated ﻿ICE VchInd5, and the SXT/R391 family [23, 74, 75], as well as the first example of a plasmid, specifically ﻿pLA6 from ﻿ *Roseobacter* [24]. Furthermore, bacterial communities from different marine regions shared a high similarity to each other [22, 25], greater than what we observed for the conjugative marine mobilome, which may be explained by an underrepresentation of MGEs diversity (e.g. 300 discrete operational units of refseq plasmid sequences) in the bacterial populations [16, 76, 77].

Our study further showed differential distribution in size (Fig. 2Table S5) and geographical dispersion between plasmids and ICEs across oceanic regions (Fig. 5A). These results are relevant because plasmid sequences are usually maintained as episomal structures in bacterial genomes and are thus prone to loss by segregation during cell division [67, 78]. In contrast, ICE sequences are integrated into the bacterial chromosome and therefore replicate as part of the chromosome, thereby reducing their loss rate [58, 79, 80]. The integrative sequences are usually located in a reduced number of hotspots and are consequently better selected and adapted to not disrupt essential host functions, thereby decreasing the metabolic burden of the host [10]. These observations, vertical inheritance and lower metabolic burden, could also explain why ICE sequences outnumbered plasmid sequences in marine bacterial genomes, especially in the larger contigs (Fig. 2B, Table S5). Moreover, this observation also coincides with a greater identification of widely dispersed ICEs in marine environments compared to plasmids [23, 24, 75]. All the above information supports the broader distribution of integrative sequences in the mobilome of marine bacterioplankton recovered in our study (Fig. 5B). Finally, in both plasmids and ICEs, the burden imposed on the host by the complete conjugative system (i.e. T4SS, T4CP, and MOB) may explain the low detection of self-conjugative sequences in marine bacterioplankton (Fig. 2A) [81]. This lower abundance of self-conjugative elements suggests high trans-acting activity (i.e. epistasis) within the marine conjugative mobilome, a mechanism already described in MGEs from other environments, such as clinical communities [16, 82].

## Conclusions

﻿To the best of our knowledge, this study is the most comprehensive analysis, to date, on the conjugative mobilome of marine bacterial communities from metagenomic data. This mobilome showed a wide geographical dispersion across oceanic regions, where MGE cargo genes were mainly related to DNA processing, with a low representation of ARGs. The results highlight the abundance of mobilizable elements in marine bacterioplankton, which outnumber self-conjugative elements among the detected sequences, suggesting a high dependence on *trans* conjugation in the mobilome sequences. The diverse MGEs marker composition and functions carried by the marine MGEs could contribute to a strategy that evens out the conjugative HGT cost–benefit balance among marine bacterial communities. This hypothesis should be studied further to aid in understanding the wide geographical dispersion that MGEs can exhibit in marine systems. Finally, in the Southern Ocean, bacterial taxa and MGEs were segregated from the rest of the oceanic regions, once again corroborating the geographic and biological isolation of this marine region.

## Supplementary Material

Tamayo-Leiva_et_al_2022_ISME_Supplementary_Material_v2_ycae059

## Data Availability

The metagenomic datasets analyzed during the current study are publicly available in the European Nucleotide Archive repository, under Project accession umber PRJEB1787 (https://www.ebi.ac.uk/ena/browser/view/PRJEB1787). Further datasets (16S rRNA miTAG) are publicly available in the TARA Oceans companion site under the following URL http://ocean-microbiome.embl.de/data/. The MAG information from the TARA Oceans metagenomes is publicly available in the Figshare repository (https://doi.org/10.6084/m9.figshare.4902923.v1).
