# Supplementary material for: Structure and dispersion of the conjugative mobilome in surface ocean bacterioplankton
Source: ISME Commun. 2024 Apr 25;4(1):ycae059. doi: 10.1093/ismeco/ycae059 (PMC11104534; doi:10.1093/ismeco/ycae059)
Supplement: Tamayo-Leiva_et_al_2022_ISME_Supplementary_Material_v2_ycae059 [file tamayo-leiva_et_al_2022_isme_supplementary_material_v2_ycae059.docx]

Supplementary Material

| **PFAM Model** | **PFAM Acc** | **Model Name** | **Function** | **Description** | **Model Length (aa)** |
| --- | --- | --- | --- | --- | --- |
| **PF01076** | PF01076.18 | Mob_Pre | Relaxase | Plasmid recombination enzyme | 196 |
| **PF02534** | PF02534.13 | T4SS-DNA_transf | T4CP | Type IV secretory system Conjugative DNA transfer | 469 |
| **PF03389** | PF03389.14 | MobA_MobL | Relaxase | MobA/MobL family | 225 |
| **PF03432** | PF03432.13 | Relaxase | Relaxase | Relaxase/Mobilisation nuclease domain | 242 |
| **PF04899** | PF04899.11 | MbeD_MobD | Relaxase | MbeD/MobD like | 70 |
| **PF05713** | PF05713.10 | MobC | Relaxase | Bacterial mobilization protein (MobC) | 45 |
| **PF07057** | PF07057.10 | TraI | Relaxase | DNA helicase TraI | 123 |
| **PF07514** | PF07514.10 | TraI_2 | Relaxase | Putative helicase | 327 |
| **PF07515** | PF07515.10 | TraI_2_C | Relaxase | Putative conjugal transfer nickase/helicase TraI C-term | 121 |
| **PF07916** | PF07916.10 | TraG_N | T4CP | TraG-like protein, N-terminal region | 467 |
| **PF08751** | PF08751.10 | TrwC | Relaxase | TrwC relaxase | 296 |
| **PF10412** | PF10412.9 | TrwB_AAD_bind | T4CP | Type IV secretion-system coupling protein DNA-binding domain | 386 |
| **PF12696** | PF12696.7 | TraG-D_C | T4CP | TraM recognition site of TraD and TraG | 126 |
| **PF13814** | PF13814.5 | Replic_Relax | Relaxase | Replication-relaxation | 192 |
| **PF16932** | PF16932.4 | T4SS_TraI | Relaxase | Type IV secretory system, conjugal DNA-protein transfer | 212 |
| **PF17511** | PF17511.1 | Mobilization_B | Relaxase | Mobilization protein B | 136 |

**Supplementary Table 1.** PFAM models for the identification of MGEs in marine microbial communities. PFAM models and accession number for the version. Type 4 coupling protein (T4CP).

| Genus | Mean | SD | Minimum | Maximum |
| --- | --- | --- | --- | --- |
| AEGEAN-169 marine group | 0.16% | 0.32% | 0.0005% | 2.40% |
| Prochlorococcus | 0.10% | 0.19% | 0.0005% | 1.78% |
| Candidatus Pelagibacter | 0.08% | 0.12% | 0.0005% | 1.04% |
| Olleya | 0.06% | 0.15% | 0.0006% | 0.55% |
| NS4 marine group | 0.05% | 0.09% | 0.0005% | 0.67% |
| Synechococcus | 0.05% | 0.12% | 0.0005% | 1.95% |
| Balneatrix | 0.05% | 0.12% | 0.0005% | 0.81% |
| NS2b marine group | 0.05% | 0.11% | 0.0006% | 0.92% |
| Marinoscillum | 0.05% | 0.08% | 0.0005% | 0.71% |
| Oleispira | 0.05% | 0.11% | 0.0007% | 0.45% |
| Arenicella | 0.05% | 0.18% | 0.0007% | 1.46% |
| Sufflavibacter | 0.05% | 0.11% | 0.0005% | 1.29% |
| Oceanobacter | 0.05% | 0.09% | 0.0007% | 0.72% |
| ZD0417 marine group | 0.04% | 0.16% | 0.0005% | 1.35% |
| Polaribacter | 0.04% | 0.16% | 0.0006% | 1.69% |
| Marinicella | 0.04% | 0.07% | 0.0005% | 0.79% |
| Cobetia | 0.04% | 0.15% | 0.0006% | 1.15% |
| Polynucleobacter | 0.03% | 0.05% | 0.0006% | 0.23% |
| Formosa | 0.03% | 0.08% | 0.0005% | 0.58% |
| NS5 marine group | 0.03% | 0.06% | 0.0005% | 0.75% |
| marine group | 0.03% | 0.11% | 0.0005% | 1.29% |
| Roseobacter clade DC5-80-3 lineage | 0.03% | 0.08% | 0.0006% | 0.60% |
| Ulvibacter | 0.03% | 0.09% | 0.0006% | 0.46% |
| Nereida | 0.03% | 0.06% | 0.0008% | 0.21% |
| Herbiconiux | 0.03% | 0.06% | 0.0007% | 0.19% |
| Oceaniserpentilla | 0.03% | 0.04% | 0.0019% | 0.06% |
| SAR92 clade | 0.03% | 0.12% | 0.0005% | 2.16% |
| NS10 marine group | 0.03% | 0.04% | 0.0006% | 0.21% |
| Thiothrix | 0.03% | 0.04% | 0.0005% | 0.27% |
| NS7 marine group | 0.02% | 0.04% | 0.0006% | 0.39% |
| Unasigned | 0.04% | 0.15% | 0.0005% | 9.22% |
| Archaea | 2.6727E-05% |  |  |  |
| Other Bacteria | 0.002% |  |  |  |

**Supplementary Table 2.** Genus level top 30 percentage abundance. Percentage abundance of the top 30 genera on the 45 stations from the TARA Oceans expedition, size fraction of free-living microorganisms (0.2–3 μm) at the surface (SRF) layer (5 m depth). Results are shown as total mean, Standard deviation, Minimum, and Maximum observed percentage abundance. Bacterial genus with low abundances were grouped as “Other Bacteria.”

|  |  | Alpha diversity  (Shannon index) | | | | Beta diversity  (Bray-Curtis distance) | | | |
| --- | --- | --- | --- | --- | --- | --- | --- | --- | --- |
| Factor | **Df** | **SS** | **R2** | **F** | **Pr(>F)** | **SS** | **R2** | **F** | **Pr(>F)** |
| Ocean and Sea regions | 5 | **0.0058157** | **0.53652** | **14.9671** | **0.0003** *** | **1.7479** | **0.37582** | **9.7197** | **0.001** *** |
| Current | 8 | 0.0011543 | 0.10649 | 1.8567 | 0.2086 | 1.1214 | 0.24111 | 3.8973 | 0.001 *** |
| Marine pelagic Biomes | 7 | 0.0007 | 0.06457 | 1.2867 | 0.3401 | 0.5712 | 0.12282 | 2.269 | 0.008 ** |
| Latitude | 1 | 0.0001312 | 0.0121 | 1.6883 | 0.217 | 0.104 | 0.02237 | 2.8925 | 0.012 * |
| Longitude | 1 | 0.0000006 | 0.00005 | 0.0075 | 0.9363 | 0.076 | 0.01634 | 2.1135 | 0.053· |
| Depth | 1 | 0.0000242 | 0.00223 | 0.3116 | 0.535 | 0.0251 | 0.0054 | 0.6978 | 0.597 |
| Temperature | 1 | 0.0012427 | 0.11464 | 15.9904 | 0.0018 ** | 0.1448 | 0.03113 | 4.026 | 0.003 ** |
| Salinity | 1 | 0.0003772 | 0.0348 | 4.8541 | 0.0482 * | 0.0559 | 0.01201 | 1.5531 | 0.124 |
| Oxygen | 1 | 0.0000034 | 0.00031 | 0.0434 | 0.8339 | 0.0437 | 0.00939 | 1.2137 | 0.274 |
| Nitrates | 1 | 0.0000185 | 0.00171 | 0.2386 | 0.6256 | 0.0765 | 0.01645 | 2.1268 | 0.044 * |
| NO2 | 1 | 0.0000023 | 0.00021 | 0.0295 | 0.8608 | 0.0489 | 0.01052 | 1.36 | 0.205 |
| PO4 | 1 | 0.0000036 | 0.00033 | 0.0458 | 0.8201 | 0.079 | 0.01698 | 2.1954 | 0.053· |
| NO2NO3 | 1 | 0.0000089 | 0.00082 | 0.114 | 0.7363 | 0.0452 | 0.00972 | 1.2575 | 0.252 |
| SI | 1 | 0.000347 | 0.03201 | 4.465 | 0.0703· | 0.0438 | 0.00942 | 1.2183 | 0.248 |
| Residual | 13 | 0.0010103 | 0.0932 |  |  | 0.4676 | 0.10053 |  |  |
| Total | 44 | 0.0108398 | 1 |  |  | 4.651 | 1 |  |  |

**Supplementary Table 3.** PERMANOVA analysis on the species variation and community structure (16S) from the selected surface stations in the TARA Oceans expedition. A permutational multivariate analysis of variance (PERMANOVA) for marginal effect on the 45 stations from the TARA Oceans expedition, size fraction of free-living microorganisms (0.2–3 μm) at the surface (SRF) layer (5 m depth). PERMANOVA test (Anderson 2001) was performed with the function adonis2 of the R package Vegan (9.999 permutations) (Oksanen *et al.,* 2019). All numerical Oceanographic variables were standardized using the z-score method (mean 0, variance 1). The Rare taxa high weights effect was standardized by Hellinger transformation in ASVs clean count data before beta diversity analysis (i.e., Bray-Curtis distance). Results are shown as alpha diversity (Shannon index) and beta diversity (Bray-Curtis distance) independent analysis. Significance codes: 0.0001 “***”, 0.001 “**”, 0.01 “*”, 0.05 “·”. The higher proportion of the variance explained by a variable is highlighted in bold for both analyses.

| Sequences Size | Number of Sequences (NR) | Number of Sequences (MAGs)* |
| --- | --- | --- |
| < 1 kb | 111 | 76 |
| 1 – 5 kb | 107 | 75 |
| 5 – 10 kb | 27 | 33 |
| 10 – 15 kb | 19 | 20 |
| 15 – 50 kb | 35 | 44 |
| 50 – 200 kb | 11 | 29 |
| > 200 kb | 3 | 8 |
| Total | 313 | 285 |

**Supplementary Table 4:** MGE sequences by size and classification. * The total number of sequences after clustering with MAGs (CD-Hit (Fu *et al.* 2012): 95% identity; 90% coverage): Collection of MAGs performed by the study of Delmont *et al.* (2018)

| Classification | Type | 1-5kb | 5-10kb | 10-15kb | 15-50kb | 50-200kb | >200kb |
| --- | --- | --- | --- | --- | --- | --- | --- |
| Conjugative | ICE | 0 | 0 | 0 | 1 | 1 | 2 |
| Conjugative | Plasmid | 0 | 0 | 1 | 0 | 3 | 0 |
| Mobilizable | ICE | 0 | 4 | 5 | 16 | 12 | 5 |
| Mobilizable | Plasmid | 70 | 17 | 11 | 7 | 11 | 1 |
| Non-conjugative | ICE | 0 | 0 | 1 | 2 | 1 | 0 |
| Non-conjugative | Secretion | 5 | 12 | 2 | 18 | 1 | 0 |
| Total |  | 75 | 33 | 20 | 44 | 29 | 8 |

**Supplementary Table 5:** MGE size distribution by mobilization and MGE type. MGE sequences size distribution by MGE mobilization type and MGE type.

| Sequence ID | Classification | Type | Lenght (kb) | MOB | Int | T4CP | ATPase | PFAM Models | Phylum | Class | Order | Family | ACC_NUCCORE | Dist |
| --- | --- | --- | --- | --- | --- | --- | --- | --- | --- | --- | --- | --- | --- | --- |
| MGE_016 | Non-conjugative | Secretion | 32.194 | - | - | + | + | Helicase HerA (DUF87) |  |  |  |  |  |  |
| MGE_019 | Non-conjugative | Secretion | 9.441 | - | - | + | - | Helicase HerA (DUF87) |  |  |  |  |  |  |
| MGE_020 | Non-conjugative | Secretion | 16.349 | - | - | + | - |  |  |  |  |  |  |  |
| MGE_021 | Non-conjugative | Secretion | 5.919 | - | - | + | - |  | Proteobacteria | Gammaproteobacteria | Pseudomonadales | Pseudomonadaceae | NZ_CP030914.1 (pY89) | 30% |
| MGE_056 | Non-conjugative | Secretion | 43.525 | - | - | + | - | UvrD-helicase | Proteobacteria | Gammaproteobacteria | Enterobacterales | Morganellaceae | NZ_KY433363.1 (pR997) | 6% |
| MGE_058 | Non-conjugative | Secretion | 2.049 | - | - | + | + |  |  |  |  |  |  |  |
| MGE_061 | Non-conjugative | Secretion | 1.315 | - | - | + | - |  |  |  |  |  |  |  |
| MGE_068 | Non-conjugative | Secretion | 8.283 | - | - | + | - | Helicase HerA (DUF87) | Firmicutes | Bacilli | Bacillales | Staphylococcaceae | NC_021230.1 (pSA8589) | 30% |
| MGE_069 | Non-conjugative | Secretion | 15.467 | - | - | + | - |  |  |  |  |  |  |  |
| MGE_090 | Non-conjugative | Secretion | 12.049 | - | - | + | - | Transposase IS116/IS110/IS902 (Transposase_20) |  |  |  |  |  |  |
| MGE_106 | Non-conjugative | Secretion | 18.088 | - | - | + | - |  |  |  |  |  |  |  |
| MGE_121 | Non-conjugative | Secretion | 30.024 | - | - | + | - | Helicase HerA (DUF87), Phage_int_SAM_2, Transposase_mut, Integrase_1, Helicase HerA (DUF87) |  |  |  |  |  |  |
| MGE_128 | Non-conjugative | ICE | 21.547 | - | + | + | + | Viral_helicase1 |  |  |  |  |  |  |
| MGE_143 | Non-conjugative | Secretion | 45.889 | - | - | + | - | UvrD-helicase | Proteobacteria | Gammaproteobacteria | Enterobacterales | Morganellaceae | NZ_KY433363.1 (pR997) | 7% |
| MGE_144 | Non-conjugative | Secretion | 8.574 | - | - | + | - |  |  |  |  |  |  |  |
| MGE_148 | Non-conjugative | Secretion | 23.765 | - | - | + | + | Helicase HerA (DUF87) |  |  |  |  |  |  |
| MGE_156 | Non-conjugative | Secretion | 8.398 | - | - | + | - |  |  |  |  |  |  |  |
| MGE_161 | Non-conjugative | Secretion | 5.987 | - | - | + | + | Helicase HerA (DUF87) |  |  |  |  |  |  |
| MGE_168 | Non-conjugative | Secretion | 7.437 | - | - | + | + | Helicase HerA (DUF87) |  |  |  |  |  |  |
| MGE_173 | Non-conjugative | Secretion | 28.698 | - | - | + | - |  | Proteobacteria | Gammaproteobacteria | Vibrionales | Vibrionaceae | NZ_CP009357.1 (p123) | 5% |
| MGE_183 | Non-conjugative | Secretion | 24.669 | - | - | + | + | Helicase HerA (DUF87) |  |  |  |  |  |  |
| MGE_190 | Non-conjugative | Secretion | 5.62 | - | - | + | + |  | Proteobacteria | Gammaproteobacteria | Enterobacterales | Enterobacteriaceae | NZ_CP044110.1 (plasmid unnamed1) | 18% |
| MGE_201 | Non-conjugative | Secretion | 43.688 | - | - | + | - | UvrD-helicase, Helicase_C, Viral_helicase1 | Actinobacteria | Actinobacteria | Micrococcales | Microbacteriaceae | NZ_CP047174.1  (plasmid unnamed1) | 30% |
| MGE_219 | Non-conjugative | Secretion | 15.802 | - | - | + | + | Helicase HerA (DUF87) |  |  |  |  |  |  |
| MGE_231 | Non-conjugative | Secretion | 8.875 | - | - | + | + | Helicase HerA (DUF87) |  |  |  |  |  |  |
| MGE_236 | Non-conjugative | Secretion | 2.998 | - | - | + | + |  |  |  |  |  |  |  |
| MGE_261 | Non-conjugative | ICE | 14.971 | - | + | + | - |  |  |  |  |  |  |  |
| MGE_266 | Non-conjugative | Secretion | 6.116 | - | - | + | + |  |  |  |  |  |  |  |
| MGE_277 | Non-conjugative | Secretion | 2.487 | - | - | + | + | Helicase HerA (DUF87) |  |  |  |  |  |  |
| MGE_282 | Non-conjugative | ICE | 25.398 | - | + | + | - | Mrr_cat; ResIII; Phage_integrase |  |  |  |  |  |  |
| MGE_288 | Non-conjugative | Secretion | 21.875 | - | - | + | + | Helicase HerA (DUF87) |  |  |  |  |  |  |
| MGE_295 | Non-conjugative | Secretion | 40.997 | - | - | + | + | Helicase HerA (DUF87) |  |  |  |  |  |  |
| MGE_296 | Non-conjugative | Secretion | 20.953 | - | - | + | + | Helicase HerA (DUF87) |  |  |  |  |  |  |
| MGE_MAG_006 | Non-conjugative | Secretion | 3.364 | - | - | - | - |  |  |  |  |  |  |  |
| MGE_MAG_012 | Non-conjugative | Secretion | 10.787 | - | - | + | - |  | Proteobacteria | Gammaproteobacteria | Legionellales |  |  | ** |
| MGE_MAG_026 | Non-conjugative | Secretion | 20.92 | - | - | + | - | Helicase_C | Firmicutes | Bacilli | Lactobacillales | Streptococcaceae | NC_004960.1 (pSRQ800) | 30% |
| MGE_MAG_030 | Non-conjugative | Secretion | 7.7 | - | - | + | + |  | Bacteroidetes | Flavobacteriia | Flavobacteriales |  |  | ** |
| MGE_MAG_031 | Non-conjugative | Secretion | 31.192 | - | - | + | - |  | Proteobacteria | Gammaproteobacteria | Legionellales |  |  | ** |
| MGE_MAG_035 | Non-conjugative | Secretion | 178.337 | - | - | + | + | DNA_gyraseA_C, DNA_gyraseB, DNA_topoisoIV, Helicase HerA (DUF87), ResIII | Proteobacteria | Deltaproteobacteria |  |  |  | ** |
| MGE_MAG_053 | Non-conjugative | Secretion | 16.465 | - | - | + | + |  | Proteobacteria | Alphaproteobacteria | Rhodobacterales |  |  | ** |
| MGE_MAG_055 | Non-conjugative | ICE | 61.194 | - | + | + | - | Phage_int_SAM_1, Phage_int_SAM_4, ResIII, Helicase_C, Integrase_1, Phage_int_SAM_2 | Proteobacteria | Gammaproteobacteria | Alteromonadales_3 | Haliea | NZ_CP019045.1 (pRR3-57) | 19% |
| MGE_MAG_061 | Non-conjugative | Secretion | 9.489 | - | - | + | - |  | Planctomycetes | Planctomycetia | Planctomycetales |  |  | ** |

**Supplementary Table 6**: Non-conjugative Genetic Elements in the ocean microbiome. Total number of type 4 secretion system genes (T4SS Genes). Number of relaxase genes (Relaxase). Number of type 4 coupling proteins (T4CP). T4SS genes (exclude relaxases and T4CP). Other putative nucleases detected in the contig (Other nucleases). ** Taxonomy assigned with TARA MAGs collection Delmont et al., 2018.

| **Sequence ID** | **Classification** | **Type** | **Lenght (kb)** | **MOBscan** | **PFAM Models Name** | **CDS** | **Phylum** | **Class** | **Order** | **Family** | **PLSDB Reference** | **Accession ID** | **Dist** | |
| --- | --- | --- | --- | --- | --- | --- | --- | --- | --- | --- | --- | --- | --- | --- |
| **MGE_192** | Conjugative | Plasmid | 14.9 | MOBP1 | Relaxase | 3 (M),9 (T), 10 (A) |  |  |  |  |  |  | |  |
| **MGE_210** | Conjugative | ICE | 47.361 | MOBP1 | Relaxase, MobC | 17,18 (M), 34 (T), 33 (A), 14 (Tyr) | Cyanobacteria |  | Nostocales | Nostocaceae | pNPUN03 | NC_010630.1 | | 29.6% |
| **MGE_MAG_014** | Conjugative | ICE | 112.332 | MOBP1 | Relaxase, MobC | 74,75 (M), 91 (T), 90 (A), 68 (Ser), 69 (Tyr) | Proteobacteria |  |  |  |  |  | | ** |
| **MGE_MAG_047** | Conjugative | Plasmid | 88.389 | MOBF | TrwC | 54 (M), 62 (T), 46 (A) | Verrucomicrobia | Opitutae | Opitutales | Hydrogenothermaceae | Unnamed | NC_012439.1 | | 29.6% |
| **MGE_MAG_045** | Conjugative | Plasmid | 55.785 | MOBless | T4SS_TraI | 26 (M), 19 (T), 27 (A) | Proteobacteria | Gammaproteobacteria | Legionellales | Piscirickettsiaceae | Ps2192A-p8 | NZ_CP050946.1 | | 29.6% |
| **MGE_MAG_041** | Conjugative | ICE | 536.733 | MOBT | T4SS_TraI | 22 (M), 231 (T), 23 (A), 386 (Ser), 5, 12, 366, 415 (Tyr) | Proteobacteria | Alphaproteobacteria |  |  | pWSZB | NZ_CP047317.1 | | 29.6% |
| **MGE_MAG_025** | Conjugative | Plasmid | 152.34 | MOBless | T4SS_TraI | 10 (M), 17 (T), 6 (A) | Proteobacteria | Gammaproteobacteria | Legionellales |  |  |  | | ** |
| **MGE_MAG_060** | Conjugative | ICE | 1,019.813 | MOBT | Replic_Relax | 327 (M), 284,325 (T), 195 (A), 75,77,301 (Ser), 314,335 (Tyr) | Proteobacteria | Gammaproteobacteria | Alteromonadales | Nocardiaceae | pDJL3 | NZ_CP025962.1 | | 29.6% |

**Supplementary Table 7:** Mobile Conjugative Genetic Elements in the ocean microbiome. Total number of type 4 secretion system genes (T4SS Genes). Number of relaxase genes (Relaxase). Number of type 4 coupling proteins (T4CP). T4SS genes (exclude relaxases and T4CP). Other putative nucleases detected in the contig and their relative position to the first CDS (CDS). Relaxase/MOB (M), T4CP (T), T4SS ATPase (A), serine integrase (Ser), tyrosine integrase (Tyr). ** Taxonomy assigned with TARA MAGs collection Delmont et al., 2018.

| **Sequence ID** | **Classification** | **Type** | **Lenght (kb)** | **Detection*** | **MOBscan** | **PFAM Models Name** | **Phylum** | **Class** | **Order** | **Family** | **PLSDB Reference** | **Accession ID** | **Distance** |
| --- | --- | --- | --- | --- | --- | --- | --- | --- | --- | --- | --- | --- | --- |
| **MGE_010** | Mobilizable | ICE | 7.046 | 7 | MOBT | Rep_trans |  |  |  |  |  |  |  |
| **MGE_021** | Mobless | Secretion | 5.919 | 15 | MOBless | NA | Proteobacteria | Gammaproteobacteria | Pseudomonadales | Pseudomonadaceae | pY89 | NZ_CP030914.1 | 30% |
| **MGE_033** | Mobilizable | Plasmid | 1.94 | 7 | MOBQ | MobA_MobL | Proteobacteria | Alphaproteobacteria | Rhodobacterales | Rhodobacteraceae | pP83_h | NZ_CP010607.1 | 22% |
| **MGE_039** | Mobilizable | Plasmid | 9.499 | 8 | MOBQ | MobA_MobL | Proteobacteria | Alphaproteobacteria | Rhodobacterales | Rhodobacteraceae | pP88_i | NZ_CP010734.1 | 22% |
| **MGE_056** | Mobless | Secretion | 43.525 | 8 | MOBless | NA | Proteobacteria | Gammaproteobacteria | Enterobacterales | Morganellaceae | pR997 | NZ_KY433363.1 | 6% |
| **MGE_092** | Mobilizable | ICE | 6.018 | 8 | MOBQ | MobA_MobL | Proteobacteria | Alphaproteobacteria | Rhodobacterales | Rhodobacteraceae | pTHAF37_g | NZ_CP045379.1 | 12% |
| **MGE_098** | Mobilizable | Plasmid | 2.196 | 8 | MOBQ | MobA_MobL | Proteobacteria | Alphaproteobacteria | Rhodobacterales | Rhodobacteraceae | pTHAF27_d | NZ_CP045397.1 | 11% |
| **MGE_141** | Mobilizable | ICE | 107.173 | 11 | MOBH | TraI_2, TraI_2_C | Proteobacteria | Gammaproteobacteria | Vibrionales | Vibrionaceae | p123 | NZ_CP009357.1 | 1% |
| **MGE_143** | Mobless | Secretion | 45.889 | 7 | MOBless | NA | Proteobacteria | Gammaproteobacteria | Enterobacterales | Morganellaceae | pR997 | NZ_KY433363.1 | 7% |
| **MGE_151** | Mobilizable | ICE | 27.449 | 6 | MOBH | TraI_2 | Proteobacteria | Gammaproteobacteria | Alteromonadales | Shewanellaceae | pS22303 | NC_011665.1 | 30% |
| **MGE_157** | Mobilizable | ICE | 29.998 | 9 | MOBH | TraI_2 | Proteobacteria | Gammaproteobacteria | Enterobacterales | Morganellaceae | pR997 | NZ_KY433363.1 | 11% |
| **MGE_161** | Mobless | Secretion | 5.987 | 7 | MOBless | NA |  |  |  |  |  |  |  |
| **MGE_172** | Mobilizable | ICE | 17.617 | 9 | MOBH | TraI_2, TraI_2_C | Proteobacteria | Gammaproteobacteria | Vibrionales | Vibrionaceae | p123 | NZ_CP009357.1 | 7% |
| **MGE_173** | Mobless | Secretion | 28.698 | 8 | MOBless | NA | Proteobacteria | Gammaproteobacteria | Vibrionales | Vibrionaceae | p123 | NZ_CP009357.1 | 5% |
| **MGE_183** | Mobless | Secretion | 24.669 | 7 | MOBless | NA |  |  |  |  |  |  |  |
| **MGE_200** | Mobilizable | Plasmid | 3.459 | 9 | MOBH | TraI_2, TraI_2_C |  |  |  |  |  |  |  |
| **MGE_201** | Mobless | Secretion | 43.688 | 20 | MOBless | NA | Actinobacteria | Actinobacteria | Micrococcales | Microbacteriaceae | punnamed1 | NZ_CP047174.1 | 30% |
| **MGE_202** | Mobilizable | Plasmid | 10.612 | 14 | MOBH | TraI_2_C, TraI_2 | Proteobacteria | Alphaproteobacteria | Rhodobacterales | Rhodobacteraceae | pTT13-6, | NZ_CP024428.1 | 30% |
| **MGE_203** | Mobilizable | Plasmid | 4.489 | 10 | MOBF | TrwC | Actinobacteria | Actinobacteria | Corynebacteriales | Mycobacteriaceae | pMSPYR102 | NC_014812.1 | 6% |
| **MGE_209** | Mobilizable | Plasmid | 3.721 | 7 | MOBP1 | Relaxase |  |  |  |  |  |  |  |
| **MGE_210** | Conjugative | ICE | 47.361 | 6 | MOBP1 | Relaxase, MobC | Cyanobacteria |  | Nostocales | Nostocaceae | pNPUN03 | NC_010630.1 | 30% |
| **MGE_231** | Mobless | Secretion | 8.875 | 8 | MOBless | NA |  |  |  |  |  |  |  |
| **MGE_266** | Mobless | Secretion | 6.116 | 7 | MOBless | NA |  |  |  |  |  |  |  |
| **MGE_268** | Mobilizable | Plasmid | 2.902 | 10 | MOBF | TrwC | Actinobacteria | Actinobacteria | Corynebacteriales | Mycobacteriaceae | pMSPYR102 | NC_014812.1 | 8% |
| **MGE_276** | Mobilizable | Plasmid | 2.62 | 7 | MOBP1, MOBQ | MobC, Relaxase | Proteobacteria | Alphaproteobacteria | Rhodobacterales | Rhodobacteraceae | pcai42A | NZ_CP012662.1 | 16% |
| **MGE_MAG_014** | Conjugative | ICE | 112.332 | 6 | MOBP1 | Relaxase, MobC | Proteobacteria |  |  |  |  |  | ** |
| **MGE_MAG_016** | Mobilizable | ICE | 16.759 | 6 | MOBC | Replic_Relax | Proteobacteria |  |  |  |  |  | ** |
| **MGE_MAG_027** | Mobilizable | ICE | 208.141 | 8 | MOBP1, MOBT | MobC | Proteobacteria | Gammaproteobacteria | Alteromonadales | Alteromonas |  |  | ** |
| **MGE_MAG_028** | Mobilizable | ICE | 55.672 | 9 | MOBF | TrwC | Proteobacteria | Gammaproteobacteria | Alteromonadales | Alteromonas |  |  | ** |
| **MGE_MAG_048** | Mobilizable | Plasmid | 6.033 | 6 | MOBF | TrwC | Actinobacteria | Actinobacteria | Actinomycetales | Microbacterium | pFQ12 | NC_002699.1 | 30% |
| **MGE_MAG_060** | Conjugative | ICE | 1019.813 | 6 | MOBT | Replic_Relax | Proteobacteria | Gammaproteobacteria | Alteromonadales |  | pDJL3 | NZ_CP025962.1 | 30% |
| **MGE_MAG_061** | Mobless | Secretion | 9.489 | 6 | MOBless | NA | Planctomycetes | Planctomycetia | Planctomycetales |  |  |  | ** |
| **MGE_MAG_064** | Mobilizable | Plasmid | 8.833 | 10 | MOBF | TrwC | Actinobacteria | Actinobacteria | Actinomycetales | Microbacterium | pA28BH2 | NZ_MN657146.1 | 30% |
| **MGE_MAG_066** | Mobilizable | Plasmid | 14.897 | 11 | MOBF | TrwC | Actinobacteria | Actinobacteria | Actinomycetales | Microbacterium | punnamed2 | NZ_CP012477.1 | 30% |

**Supplementary Table 8:** Mobile Genetic Elements with higher prevalence in the ocean microbiome. List and features of the MGEs with higher prevalence in the ocean microbiome. MGEs were classified as “detected" in a metagenome when at least the 80% of contig length reported a ≥ 1x coverage. ** Taxonomy assigned with TARA MAGs collection Delmont et al., 2018.

**
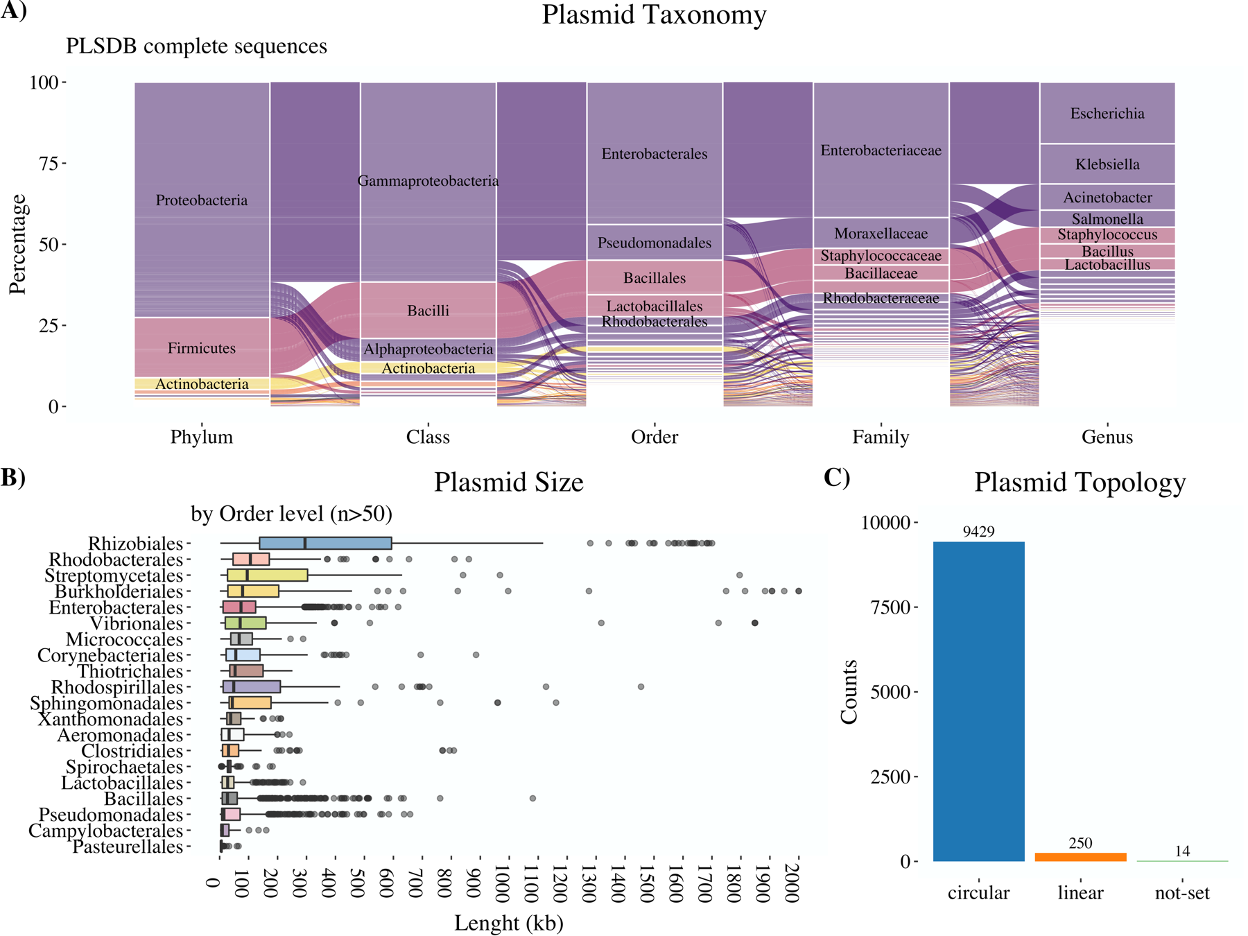
Supplementary Figure 1**. PLSDB sequences data features. **A)** Taxonomic assignation of the 9.693 PLSBD (Galata *et al.* 2018) plasmid sequences annotated as “complete”. **B)** Plasmid size of the sequences by order taxonomic level. Only Orders with 50 or more sequences are shown. **C)** Plasmid topology of all selected sequences.


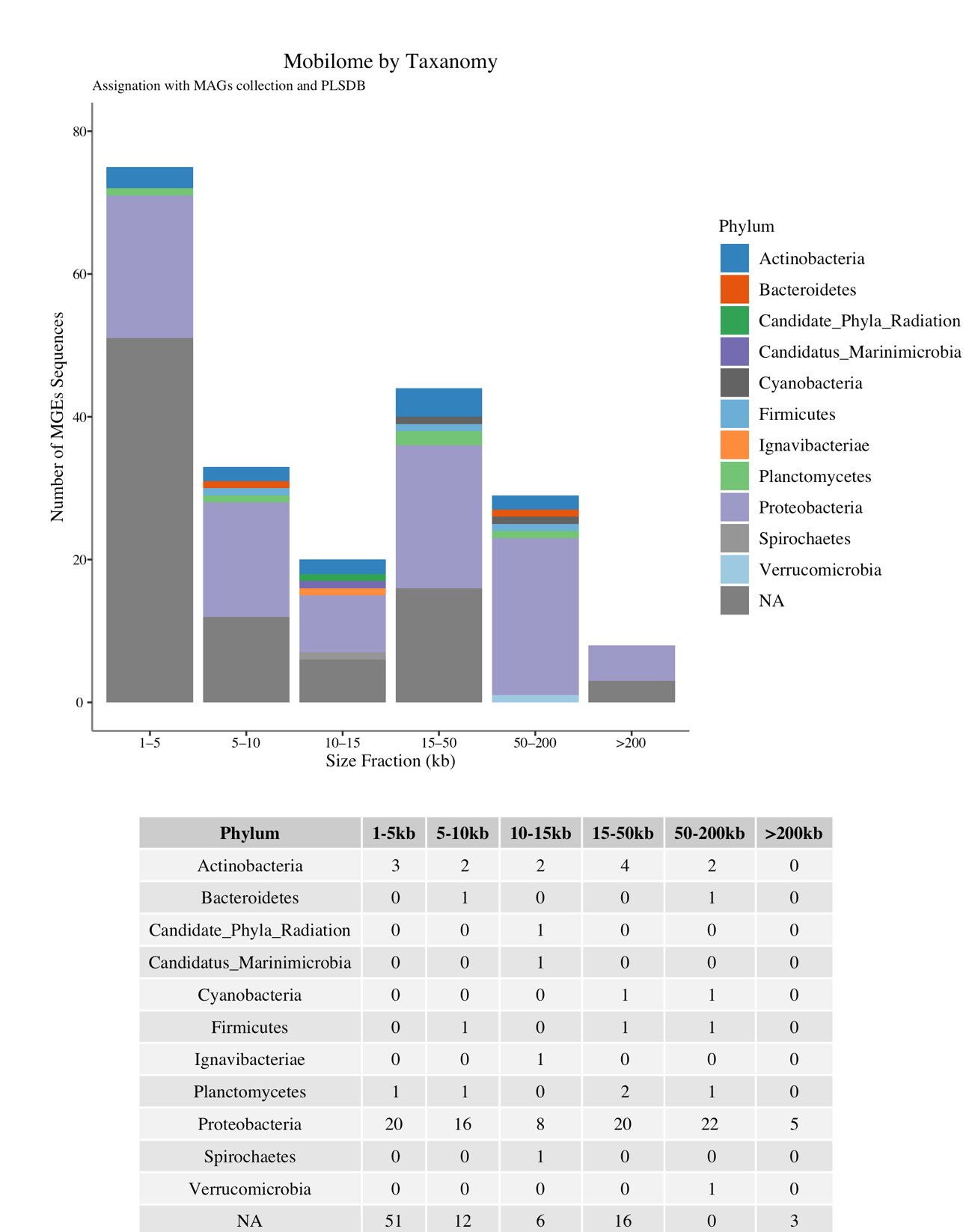
**Supplementary Figure 2**. Taxonomical identity of the 313 putative MGE non-redundant (nr) sequences. The 313 MGE (NR) sequences were compared with the collection of 957 curated metagenomes-assembled genomes (MAGs) based on TARA metagenomes co-assemblies, and reported by Delmont et al., 2018, plus the 9.693 plasmid sequences annotated as “complete” in the curated plasmid database (PLSDB) (Galata et al. 2018).

**Supplementary Figure 3**.
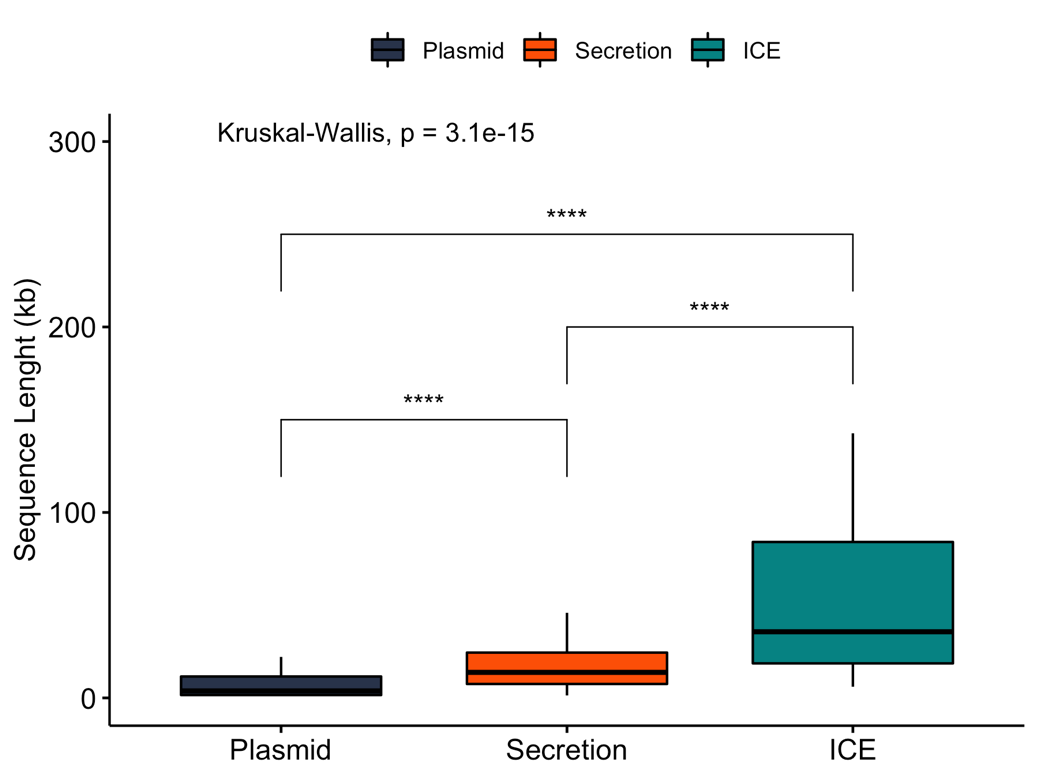
 Differential size distribution on the marine mobilome MGE types. Statistical analysis of the size distribution variance among the different types of mobile genetic elements found and classified in the study. For ease the visualization, outliers were removed from the final plot.

**Supplementary Figure 4**.
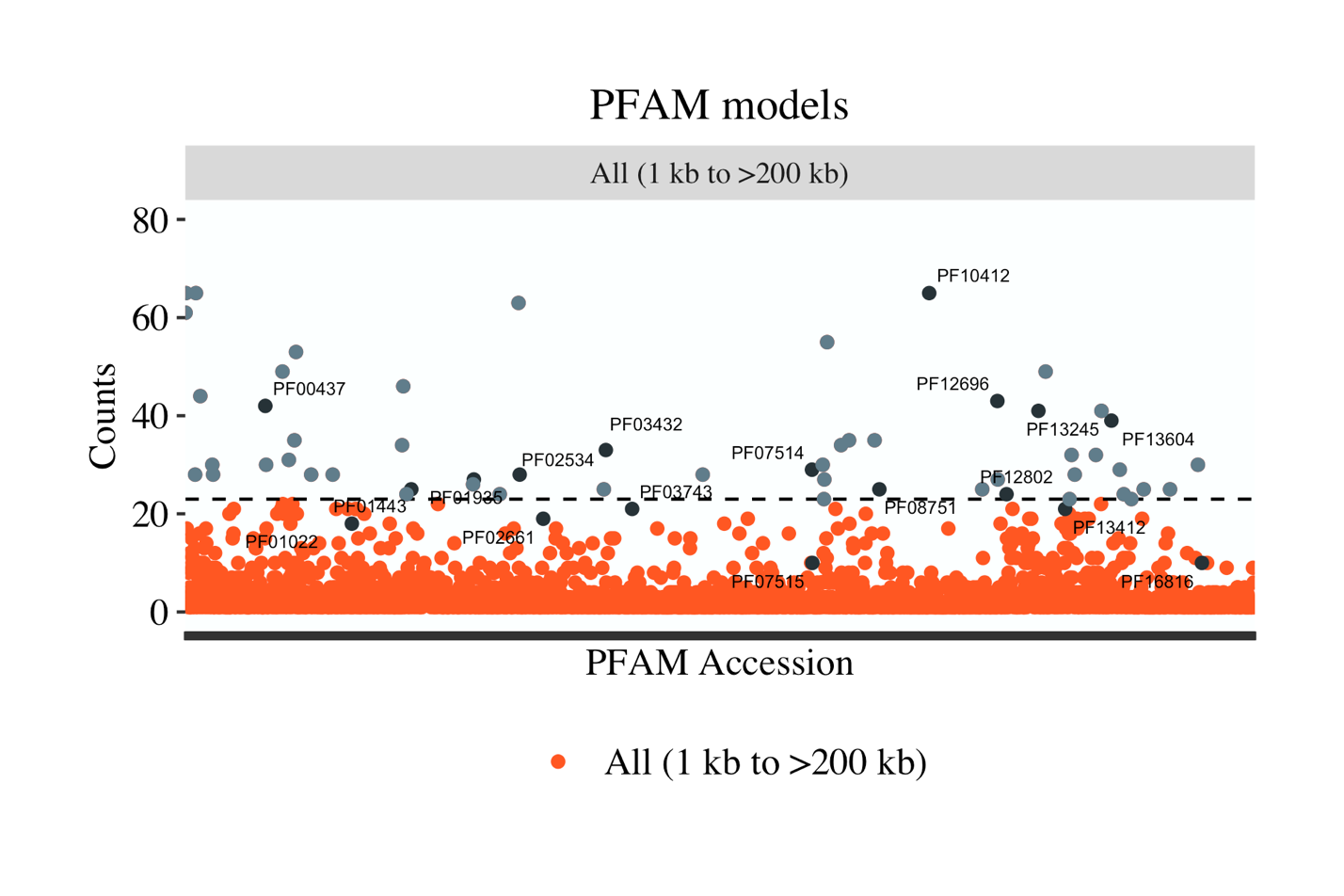
 Core and non-core PFAM models detected in the most abundant 2% of cumulative counts. Segmented black line denotes the 2% threshold. Black dots denote core PFAM models. Grey dots denote PFAM models non-core in the upper 2%. Red dots represent all the non-core PFAM models below the 2% threshold.

**
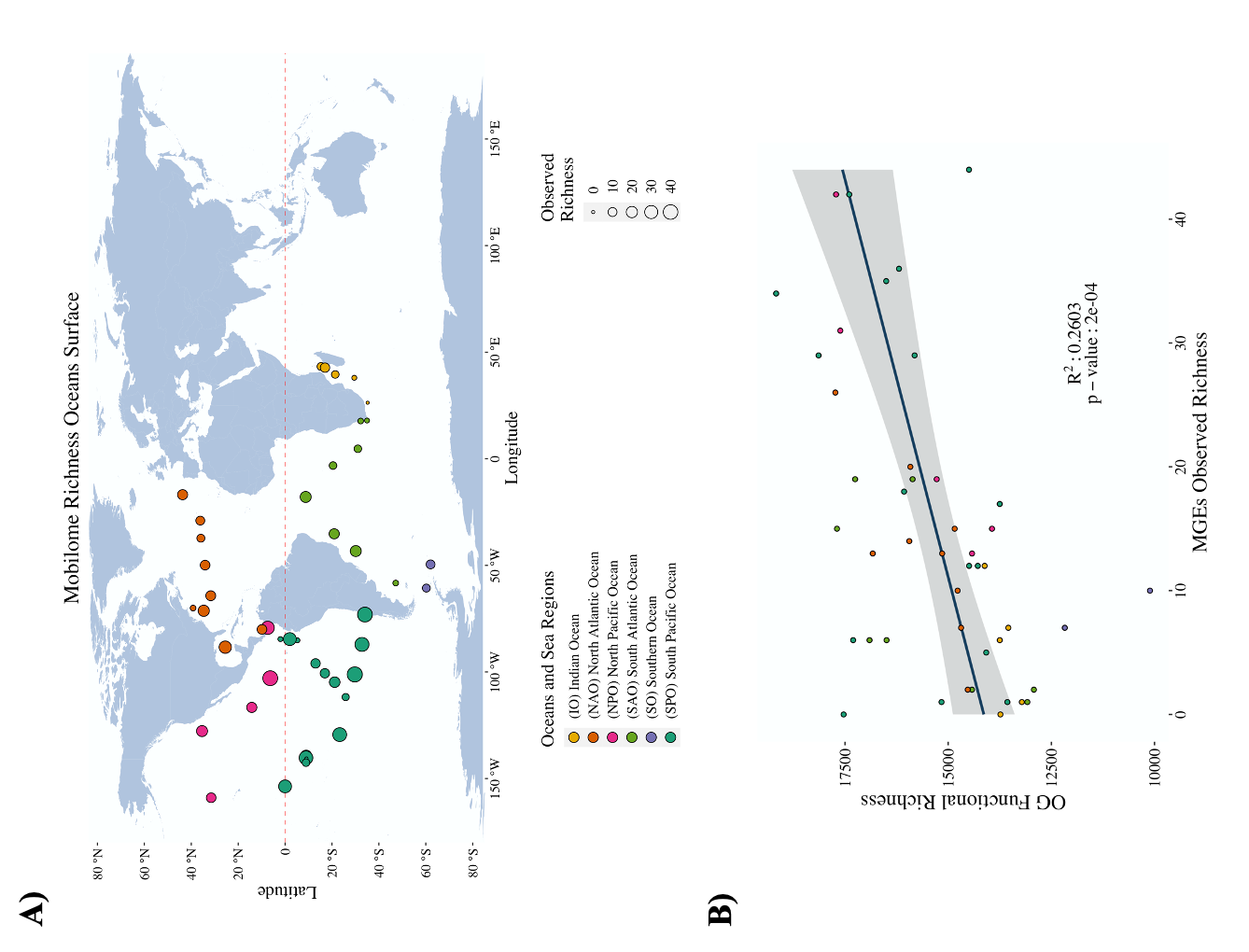
**

**Supplementary Figure 5**. Mobilome richness in surface marine waters samples. Stations selected on this study from TARA Oceans dataset. Size-fraction 0.22 - 3.0 m. A) Samples selected and enumerated by station id. Stations are colored by Ocean’s regions (SPO) South Pacific Ocean, (NAO) North Atlantic Ocean, (SO) Southern Ocean, (NPO) North Pacific Ocean, (SAO) South Atlantic Ocean, (IO) Indian Ocean. Point size represent the number of sequences detected with (80% sequence length with ≥ 1 coverage) at each sample. B) Linear model of the MGEs observed richness, number of sequences detected with (80% sequence length with ≥ 1 coverage) and the Orthologous genes functional richness acquired from the TARA Oceans dataset. Color represents each ocean and sea region.

**
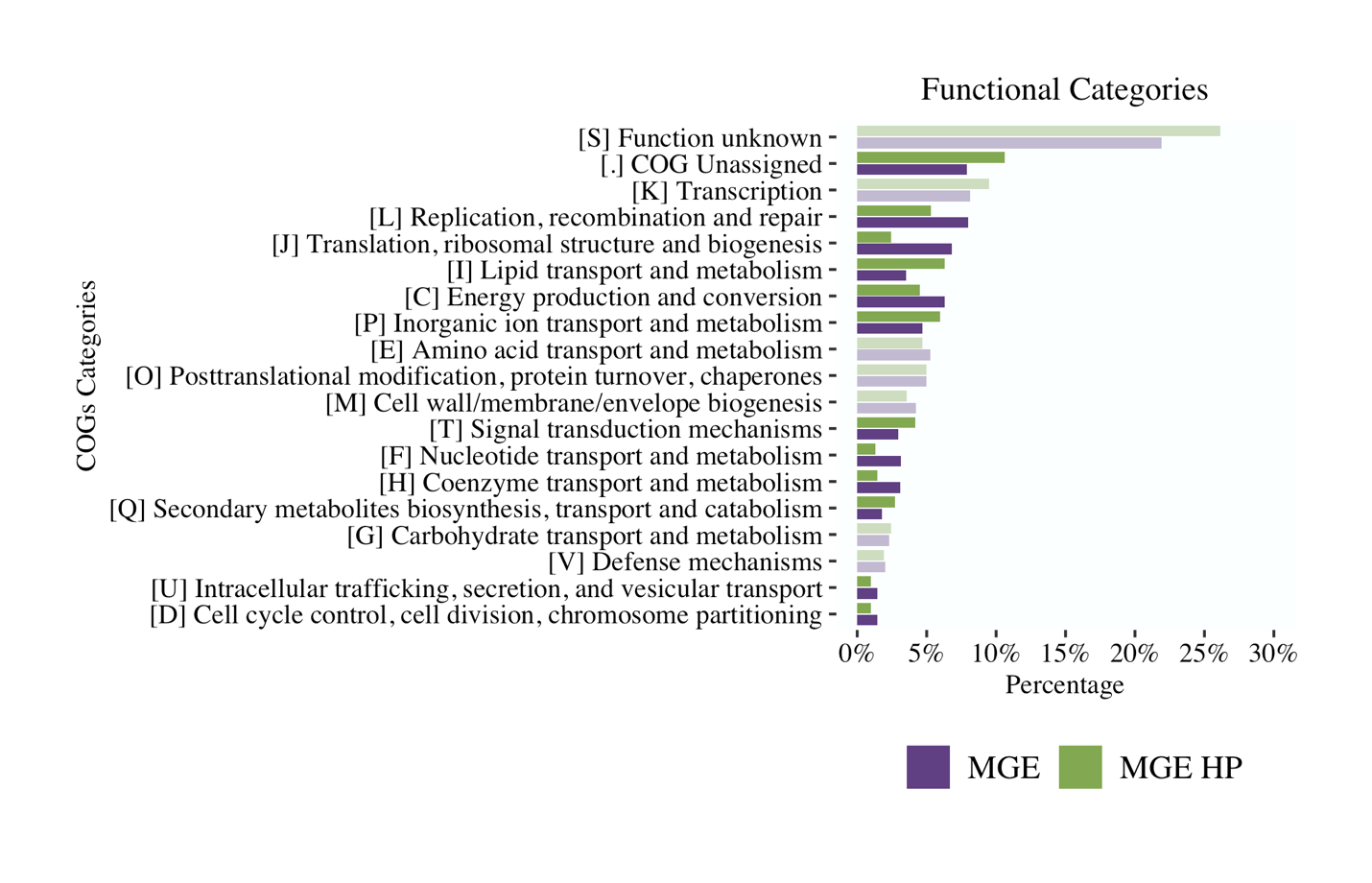
Supplementary Figure 6**. Functional COG annotation detected in the MGEs with higher prevalence (MGE HP) versus the collection of MGE in the ocean microbiome. COG functions, from MGEs sequences reported with at least the 80% of the contig length with an ≥ 1x coverage in six or more different stations from the TARA Oceans dataset are shown in green while in blue MGE collection. COG groups where the difference between groups was of the 20% or higher are shown in solid colors, if the difference was lower, the groups are show with transparency.

**References**

Anderson MJ. A new method for non-parametric multivariate analysis of variance. *Austral Ecol* 2001;**26**:32–46.

Delmont TO, Quince C, Shaiber A *et al.* Nitrogen-fixing populations of Planctomycetes and Proteobacteria are abundant in surface ocean metagenomes. *Nat Microbiol* 2018;**3**:804–13.

Fu L, Niu B, Zhu Z *et al.* CD-HIT: Accelerated for clustering the next-generation sequencing data. *Bioinformatics* 2012;**28**:3150–2.

Galata V, Fehlmann T, Backes C *et al.* PLSDB: a resource of complete bacterial plasmids. *Nucleic Acids Res* 2018;**47**:D195–202.

Oksanen J, Blanchet FG, Friendly M *et al.* vegan: Community Ecology Package. 2019.
